# Structured reporting of pelvic MRI leads to better treatment planning of uterine leiomyomas

**DOI:** 10.1007/s00330-018-5417-z

**Published:** 2018-05-04

**Authors:** Evis Sala, Susan Freeman

**Affiliations:** Department of Radiology, Box 218, Cambridge Biomedical Campus, Cambridge, CB2 0QQ UK

Over the years, the role of the radiologist within the multidisciplinary team has evolved remarkably, with imaging providing crucial information for patient management. Through close collaboration with referring clinicians, most radiology practices now strive for their radiology reports to provide the maximum value for individualized patient care [1]. Therefore, the development of structured radiology reports has gained impetus as an essential tool toward delivering personalized medicine. In fact, structured report templates provide a platform for potentially providing clear, concise, consistent and actionable reports that can assist the referring clinician in triaging the patient to appropriate treatment [1]. The key to adding value to radiology reporting lies in the disease-specific structured reports that are developed by radiologists in collaboration with the clinical management team. However, in the era of increasing workload, the balance between a succinct, generic structured report and a time-consuming disease-specific report is important.

In general, structured reporting allows information to be more easily extracted and improves communication with clinicians. In addition, the use of structured reporting reduces ambiguous terms and errors due to use of speech recognition systems, typically seen with narrative reports, which could lead to misinterpretation and in turn impact patient management [2]. Furthermore, standardized template reporting enhances the value of natural language processing and machine learning techniques, which have been shown to successfully extract relevant prognostic information from radiology reports [3]. The clarity of the radiology report is essential to the integration of imaging, pathology, multi-omics and clinical data and provides one of the cornerstones of integrative and personalized medicine.

Aiming at enhancing the quality and efficiency of radiology reports, the Radiology Society of North America (RSNA) and the European Society of Radiology (ESR) have jointly formed the Template Library Advisory Panel (TLAP) [4]. TLAP provides reporting templates that are based on established data standards and incorporate structured terminology such as RSNA RadLex radiology lexicon, as well as access to tools to create and modify templates. The overall goal is to improve the value of the radiology service to patients and their treating physicians by providing consistent and data-rich reports, which also enable better data analysis for outcomes research when compared to narrative unstructured reports.

Several studies have analyzed the value of structured reporting, most of them focusing on various oncological applications [5–7]. Brook and colleagues showed that implementation of structured reports leads to improvement in staging and surgical planning in patients with pancreatic cancer [5]. Yee and colleagues developed a computed tomography (CT) colonography structured reporting template that led to improved clarity of interpretation and thus better communication with the referring physicians [6]. In patients with primary rectal cancer, structured rectal magnetic resonance imaging (MRI) reporting templates facilitated surgical planning and led to higher satisfaction level of referring surgeons compared to narrative reports [7]. Other studies assessed the structured format of the conclusion/impression section of the report as a means to provide better value to the managing team [8, 9]. Introduction of a structured format in the impression section of a coronary CT angiography report led to an improved agreement on the number of significant stenotic vessels [8]. More recently, Wibmer and colleagues [9] found that the implementation of a lexicon of diagnostic certainty in prostate MRI reporting significantly reduced the number of expressions used by radiologists to indicate their levels of diagnostic certainty for presence of extracapsular extension. This is important as wider adoption of such a lexicon might prevent miscommunication and help referring clinicians to reliably incorporate radiologists’ input into clinical decision-making.

The study by Franconeri and colleagues [10] investigated the clarity and impact of structured reporting, in comparison to narrative reporting, on treatment planning for patients with uterine leiomyomas. The most important and novel aspect of the study design is that the structured reporting template was developed by diagnostic radiologists in collaboration with interventional radiologists and gynecologists. The reports were assessed both objectively and subjectively by the same multidisciplinary team; this is another major strength of the study design, as patient management decisions in clinical practice are made by multidisciplinary teams. This is key to successful implementation and acceptance of such reports by the management team and ultimately impacts individual patient care. The authors also found that structured reports described the key features of uterine leiomyomas and provided sufficient information to enable treatment planning more frequently, when compared to narrative reports. More importantly, structured reports were more helpful for surgical planning and easier to understand by gynecologists compared to narrative reports.

Template reporting improves the consistency of radiology reports and assures a terminology accepted by everyone involved in patient care. Selection of the clinically important features that determine patient management and treatment selection is crucial and best done in consultation with the clinical management team. In fact, the study by Franconeri and colleagues [10] proves that structured reports developed in consultation with treating gynecologists and interventional radiologists reduce the number of missing key features, are easier to understand and more likely to contain sufficient information for procedural planning in patients with uterine leiomyomas. One could argue that in the era of proliferation of multidisciplinary team meetings (MDTs), they will provide the best platform to discuss overall a patient’s clinical and imaging information. However, MDTs are increasingly time-consuming and usually focused on patients on oncology pathways. Therefore, the use of disease-specific structured reporting for both oncological and non-oncological applications might reduce the number of patients discussed at MDTs. Consequently, this will allow more time for adequate discussion of complicated cases as well providing a better educational forum.
